# Comparing Message-Based Psychotherapy to Once-Weekly, Video-Based Psychotherapy for Moderate Depression: Randomized Controlled Trial

**DOI:** 10.2196/46052

**Published:** 2023-06-29

**Authors:** Jiyoung Song, Boris Litvin, Ryan Allred, Shiyu Chen, Thomas D Hull, Patricia A Areán

**Affiliations:** 1 Department of Psychology University of California, Berkeley Berkeley, CA United States; 2 Talkspace New York, NY United States; 3 Department of Psychiatry and Behavioral Sciences University of Washington Seattle, WA United States

**Keywords:** randomized controlled trial, message-based psychotherapy, video-based psychotherapy, telemedicine, depression, anxiety, functional impairment, credibility, alliance, engagement, mental health, text mining, message therapy, Burden of Disease, telehealth, intervention

## Abstract

**Background:**

Despite the high prevalence of major depressive disorder and the related societal burden, access to effective traditional face-to-face or video-based psychotherapy is a challenge. An alternative that offers mental health care in a flexible setting is asynchronous messaging therapy. To date, no study has evaluated its efficacy and acceptability in a randomized controlled trial for depression.

**Objective:**

The aim of this study was to compare the efficacy and acceptability of message-based psychotherapy for depression to once-weekly video-based psychotherapy.

**Methods:**

In this 2-armed randomized controlled trial, individuals (N=83) with depressive symptomatology (Patient Health Questionnaire-9 ≥10) were recruited on the internet and randomly assigned to either a message-based intervention group (n=46) or a once-weekly video-based intervention group (n=37). Patients in the message-based treatment condition exchanged asynchronous messages with their therapist following an agreed-upon schedule. Patients in the video-based treatment condition met with their therapist once each week for a 45-minute video teletherapy session. Self-report data for depression, anxiety, and functional impairment were collected at pretreatment, weekly during treatment, at posttreatment, and at a 6-month follow-up. Self-reported treatment expectancy and credibility for the assigned intervention were assessed at pretreatment and therapeutic alliance at posttreatment.

**Results:**

Findings from multilevel modeling indicated significant, medium-to-large improvements in depression (*d*=1.04; 95% CI 0.60-1.46), anxiety (*d*=0.61; 95% CI 0.22-0.99), and functional impairment (*d*=0.66; 95% CI 0.27-1.05) for patients in the message-based treatment condition. Changes in depression (*d*=0.11; 95% CI –0.43 to 0.66), anxiety (*d*=–0.01; 95% CI –0.56 to 0.53), and functional impairment (*d*=0.25; 95% CI –0.30 to 0.80) in the message-based treatment condition were noninferior to those in the video-based treatment condition. There were no significant differences in treatment credibility (*d*=–0.09; 95% CI –0.64 to 0.45), therapeutic alliance (*d*=–0.15; 95% CI –0.75 to 0.44), or engagement (*d*=0.24; 95% CI –0.20 to 0.67) between the 2 treatment conditions.

**Conclusions:**

Message-based psychotherapy could present an effective and accessible alternative treatment modality for patients who might not be able to engage in traditional scheduled services such as face-to-face or video-based psychotherapy.

**Trial Registration:**

ClinicalTrials.gov NCT05467787; https://www.clinicaltrials.gov/ct2/show/NCT05467787

## Introduction

Major depressive disorder, a highly prevalent mental health condition characterized by persistent negative mood and loss of pleasure, is linked to significantly decreased quality of life and poses a major societal burden [1]. According to the most recent Global Burden of Diseases study [2], major depression was the 4th leading cause of disability in the 10-24-year age group and the 13th leading cause across all ages. Overall, 8.7% of the adult United States population was estimated to meet the clinical diagnosis of major depressive disorder between 2017 and 2018, and this rate has dramatically increased to 14.4%-27.8% since the beginning of the COVID-19 pandemic in early 2020 [3,4]. Higher risks of such elevated depressive symptoms were linked to lower income (defined as having less than US $5000 in savings) and greater pandemic-related stressors, attesting to the connection between low socioeconomic status and major depression.

Telehealth interventions present especially attractive options for alleviating such mental health disparities [5]. Telehealth interventions not only put a less financial burden on patients than in-person interventions but can also reach places where traditional mental health services are scarce [6]. Recent research has addressed the potential for telehealth interventions to treat patients with major depression and other mental health disorders. Even before the COVID-19 pandemic, over 10,000 mobile mental and behavioral health apps were available for use, rendering the need for further research and evaluation of their efficacy given the range of reviews for these apps [7]. In the context of social distancing and other changes enforced by the COVID-19 pandemic, the use of telehealth options by psychologists rose from 7.1% before the pandemic to 85.5%, marking the absolute necessity of telehealth interventions during this time [8].

Among the various forms of telehealth interventions, asynchronous message-based psychotherapy holds particular promise, as it is more likely to be accessible through mobile apps and is met with less skepticism as a means of intervention [9]. Unlike synchronous telehealth interventions, in asynchronous message-based psychotherapy, patients can seek discreet and flexible support as needed and on their own schedule, as opposed to waiting once a week or every other week to meet with a therapist [10]. Given that patients in rural areas consistently remain underserved despite the high prevalence of mental health conditions, asynchronous message-based psychotherapy holds the potential to improve access where mobile technologies are readily available [8,11-14].

Previous studies have demonstrated that asynchronous message-based psychotherapy is acceptable and feasible for treating various mental health conditions, including major depression [15]. In a small-scale study, 30 patients and 30 therapists in asynchronous message-based psychotherapy were able to form therapeutic alliance comparable to, if not better than, face-to-face therapy [16]. Individuals receiving asynchronous message-based psychotherapy also reported satisfaction with affordability, convenience, and flexibility [17]. Similar to traditional psychotherapy, higher engagement in asynchronous message-based psychotherapy predicted better treatment outcomes, demonstrating the need for active participation by both patients and therapists for effective treatment [10]. Asynchronous message-based psychotherapy also demonstrated feasibility as an adjunct therapy combined with traditional counseling services. In a sample of majority non-Hispanic African American mothers of low socioeconomic status, patients described asynchronous message-based psychotherapy as a positive means of motivation to change and address depressive symptoms [18].

Asynchronous message-based psychotherapy also appears to be efficacious in improving depressive symptoms. The results of a pilot study assessing message-based asynchronous psychotherapy treatment over 12-16 weeks demonstrated that patients reported significantly less life distress following 3.9 months of treatment, with 46% experiencing clinically significant symptom remission [16]. In another study, 84% of patients no longer met the threshold for major depression after 14-15 weeks of asynchronous message-based psychotherapy [19]. Similarly, in a large-scale study of 10,718 outpatient participants with depression and anxiety, a clinically significant change in depressive symptoms was observed in 53% of the sample. Asynchronous message-based psychotherapy has also demonstrated the potential to treat major depression among patients in underserved settings. Patients in both rural and urban cohorts reported significant improvements in their depressive symptoms; the symptom reduction was greater for the rural cohort [13].

Although existing research shows potential for treating major depression through asynchronous message-based psychotherapy, the need for further randomized controlled trials remains. The purpose of this study was to evaluate whether asynchronous message-based psychotherapy is clinically effective and acceptable for treating major depression when compared to weekly video-based psychotherapy (a standard practice) in a randomized controlled trial. Treatment efficacy was measured by changes in depression, anxiety, and functional impairment, and acceptability by treatment credibility and expectancy, therapeutic alliance, and engagement. It was hypothesized that both the treatment efficacy and acceptability of asynchronous message-based psychotherapy would be noninferior to those of video-based psychotherapy. That is, reductions in depression, anxiety, and functional impairment in the message-based condition would not be significantly smaller than those in the video-based condition. Similarly, levels of treatment credibility and expectancy, therapeutic alliance, and engagement in the message-based condition would not be significantly lower than those in the video-based condition.

## Methods

### Study Design

In this 2-armed randomized controlled trial, 83 patients were randomly assigned to either the message-based psychotherapy group or the video-based psychotherapy group. Consulting therapists screened patients for eligibility criteria by reviewing their screener assessments and conducting a structured clinical interview. The consulting therapist proceeded to consent eligible patients who expressed interest in participating in the study. Before, during, and 6 months after receiving the randomly assigned intervention, participants completed various efficacy and acceptability measures.

### Participants and Study Procedure

Between February 8, 2020, and April 27, 2020, participants were recruited on the internet through ads on the website of Mental Health America [20], a national advocacy organization for individuals looking to explore their mental health needs and treatment options. Advertisement links sent individuals to a screener assessment and brief intake with a consulting therapist. Inclusion criteria consisted of being English speakers in the United States, being 18 years old or older, having internet access via computer or smartphone, being computer literate, receiving a depression-related diagnosis by the consulting therapist, and scoring 10 or higher on the 9-item Patient Health Questionnaire (PHQ-9). During the same structured clinical interview by the consulting therapist, participants who indicated bipolar disorder, substance use disorders, schizophrenia or psychosis spectrum disorders or features, medical conditions that would better explain their condition, or active suicidal behavior requiring emergency services were excluded from the study. Participants meeting the criteria who then consented to be part of the study created accounts on the platform [21] and were randomized to their treating therapist and condition, after which treatment began.

Participants completed study assessments through web-based self-report surveys sent to participants via REDCap electronic data capture tools [22,23] hosted at the University of Washington. REDCap is a secure, web-based platform designed to support data collection and automated survey distribution. Assessments were administered at pretreatment, then on a weekly basis for 12 weeks, and again at 6 months. Measures administered at pretreatment included a sociodemographic survey, the PHQ-9, the additional impairment item from the PHQ-9, the 7-item Generalized Anxiety Scale (GAD-7), and the Expectations about Treatment/Treatment Rationale Scale (TRS). Weekly measures included the PHQ-9, the additional impairment item from the PHQ-9, and the GAD-7. At 12 weeks, in addition to the weekly measures, participants completed the Working Alliance Inventory—Short Revised (WAI-SR). At 6-month follow-up, the following measures were administered: the PHQ-9, the additional impairment item from the PHQ-9, and the GAD-7.

### Randomization and Blinding

Following consent, the consulting therapist randomized participants to either daily message-based psychotherapy or once-weekly video-based psychotherapy. Randomizations were carried out within therapists, meaning that each therapist would receive half messaging-only participants and half video-only participants. A simple randomization sheet [24] was used to generate the random allocation sequence. Intervention conditions were assigned by consulting therapists who were neither part of the research team nor providing care to participants in the trial. No information was provided to participants about which one of the 2 interventions was of interest.

### Interventions

#### Message-Based Psychotherapy

Participants in this condition exchanged asynchronous text, audio, and video messages with their therapist, who replied each day following an agreed-upon schedule. All interactions were conducted on a HIPAA (Health Insurance Portability and Accountability Act)-compliant digital mental health platform accessible through desktop computers and internet-enabled smartphones. Participants were free to send messages at any time and of any type (eg, text, audio, and video).

#### Video-Based Psychotherapy

Participants in this condition met with their therapist once each week for a scheduled 45-minute video teletherapy session on the same HIPAA-compliant digital mental health platform as for the message-based condition. Participants and therapists could use messaging for scheduling purposes, but for no other reason. All clinical work was saved for the weekly video session.

#### Therapists

The treating therapists were predominantly female (87.0%), and 80.1% of them had 5 or more years of postlicensure experience using cognitive behavioral therapy for depression. Therapists treated equal numbers of participants in both conditions to reduce the impact of therapist effects on the condition. Therapists received 8-hour continuing education units in evidence-based practice for depression offered through the service.

All therapists were licensed and trained to detect, evaluate, and de-escalate crisis situations, including referring individuals to in-patient or other levels of care. Any behaviors of concern were also reported to the study team.

### Measures

#### Sociodemographic Variables

At pretreatment, participants provided sociodemographic information. The survey included questions about age, gender, race, ethnicity, income, and years of education.

#### Clinical Diagnosis

The diagnosis was determined through a combination of pretreatment patient reports, PHQ-9 and GAD-7, and a clinical interview with each participant's licensed therapist.

#### Depression

To assess depression severity over the course of treatment, participants completed the PHQ-9 [25] at pretreatment, after each session, and at posttreatment. The PHQ-9 is a widely used 9-item self-report measure that assesses the frequency and severity of depressive symptomatology in the past 2 weeks. Each item is rated on a 4-point Likert scale that ranges from 0 (not at all) to 3 (nearly every day). The PHQ-9 has demonstrated sound psychometric properties in the literature [25,26], and its multilevel internal consistencies in this study were .94 and .84 at the between-participants and within-participants levels, respectively.

#### Anxiety

To assess anxiety severity over the course of treatment, participants completed the GAD-7 [27] questionnaire at pretreatment, after each session, and at posttreatment. The GAD-7 is a widely used 7-item self-report measure that assesses the frequency and severity of anxiety symptomatology in the past 2 weeks. Each item is rated on a 4-point Likert scale that ranges from 0 (not at all) to 3 (nearly every day). The GAD-7 has demonstrated sound psychometric properties in the literature [27,28], and its multilevel internal consistencies in this study were .95 and .84 at the between-participants and within-participants levels.

#### Functional Impairment

To assess functional impairment over the course of treatment, participants completed the impairment question of the PHQ-9 [25] at pretreatment, after each session, and at posttreatment. This item asks, “If you checked off any problems, how difficult have these problems made it for you to do your work, take care of things at home, or get along with other people?” The item is rated on a 4-point Likert scale that ranges from 0 (not difficult at all) to 3 (extremely difficult). This item has demonstrated sound psychometric properties in the literature [25,26].

#### Credibility and Expectancy

Treatment credibility and expectancy are crucial components of treatment acceptability and also important predictors of treatment outcome [29-32]. To assess the treatment credibility and expectancy of participants, participants completed the TRS [33] after they were assigned either of the 2 study conditions at pretreatment. The 4 questions were: “How logical does your Talkspace treatment seem to you?” “How confident are you that the treatment will be successful in eliminating your depression?” “How confident would you be in recommending the treatment to a friend with depression?” and “If you were extremely depressed, would you be willing to undergo such treatment?” In this study, each item was rated on a 10-point Likert scale that ranges from 1 (not at all) to 10 (very). The TRS has demonstrated sound psychometric properties in the literature [34], and its internal consistency in this study was .80.

#### Therapeutic Alliance

The ability to form therapeutic alliance between the therapist and client is crucial to effective treatment [35]. To assess therapeutic alliance, participants completed the WAI-SR [36] at posttreatment. The WAI-SR is a widely used 12-item self-report measure that assesses therapeutic alliance across 3 domains: agreement on the tasks of therapy, agreement on the goals of therapy, and development of affective bond. Each item is rated on a 5-point Likert scale that ranges from 0 (seldom) to 5 (always). The WAI-SR has demonstrated sound psychometric properties in the literature [36,37], and its internal consistency in this study was .92.

#### Engagement

This study sought to examine whether there were significant differences between the 2 treatment conditions in their levels of participant engagement. The final session number attended by each participant was used to measure their engagement, ranging from 0 (dropping out before the treatment began) to 12 (completing all sessions). A week of messaging was considered a “session” for the purposes of comparing the 2 conditions.

#### Statistical Analysis

Data were analyzed using R software (version 4.2.0; R Foundation for Statistical Computing), and all available data from the participants were included in intent-to-treat analyses. Treatment outcome and acceptability variables were compared between the 2 groups across the available time points. For continuous variables (eg, PHQ-9, GAD-7), Welch *t* tests were used to account for the unequal variances between the 2 treatment conditions. For categorical variables (eg, gender, race, ethnicity), Fisher exact tests were used instead of chi-square tests given the relatively small sample size in each treatment condition.

To examine the rates of change in outcome variables over the course of treatment, multilevel models were constructed using the *nlme* and *lmerTest* packages. The *nlm*e package’s default maximum likelihood estimation handles missingness in the data set. To account for the nested structure of the data, random intercepts and slopes of time (session number) by participants were included in the models. The 95% confidence intervals for the rates of change were derived from the coefficients of time and compared between the 2 treatment conditions. Cohen *d* effect sizes were computed to represent the magnitude of between-group and within-group differences for the *t* tests and rates of change from the multilevel models, where *d*=0.20, 0.50, and 0.80 indicate small, medium, and large effect sizes, respectively.

### Ethics Approval and Consent to Participate

This study was approved by the University of Washington institutional review board (STUDY00007239). All study procedures adhered to the guidance listed in the latest version of the Declaration of Helsinki. Eligible participants who expressed interest in the study were given the opportunity to ask questions during the consent process, and those who agreed to participate in the study provided written informed consent. Data provided by participants were stored on a secure server hosted at the University of Washington, and only the deidentified data were analyzed for this study.

Participants were paid US $100 for completing all weekly surveys during the 12 weeks of study treatment and an additional US $70 for completing the 6-month follow-up survey, for a total of up to US $170. Participants were paid via digital gift cards (Amazon eGift Cards).

## Results

### Sample Characteristics

**Figure 1:**
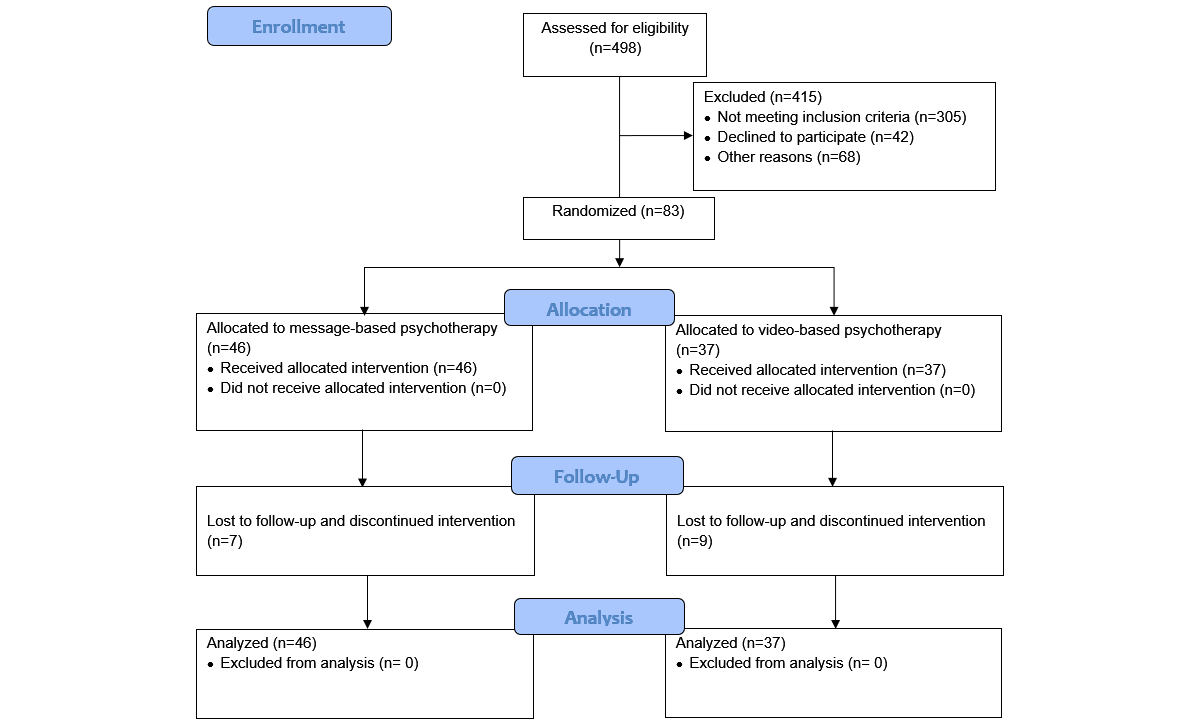
CONSORT (Consolidated Standards of Reporting Trials) flow diagram.

The CONSORT (Consolidated Standards of Reporting Trials) flow diagram is shown in Figure 1, and demographic information for the 2 treatment conditions is included in Table 1. On average, participants (N=83) were 28.66 (SD 11.48) years old, and the majority were female (51/83, 61%). Ten participants (10/83, 12%) identified as Asian, 7 (7/83, 8%) as Black, 1 (1/83, 1%) as Native American, 28 (28/83, 34%) as White, and 3 (3/83, 4%) as multiracial. Most participants were non-Hispanic (42/83, 51%). Thirty-four (34/83, 41%) participants made less than US $25,000 a year. The mean number of years of education for the whole sample was 14.95 (SD 2.28). The 2 treatment conditions did not significantly differ in these demographic variables except for the years of education. Participants in the video treatment condition (mean 15.68, SD 2.15) completed a significantly higher number of years of education than those in the message treatment condition (mean 14.43, SD 2.25; *t*_53.2_=2.18, *P*=.03). At pretreatment, participants in both treatment conditions reported moderate levels of depression (mean 16.55, SD 5.34), anxiety (mean 12.21, SD 4.99), and functional impairment (mean 1.81, SD 0.86). The 2 groups did not significantly differ in any of the 3 outcome variables at pretreatment. No harm or unintended effects were found in either group.

Our final sample size had 80% power to be sure that the lower limit of a 1-sided 95% CI would be above the noninferiority limit of a small to medium effect (Cohen *d*=0.44).

**Table 1:** Participant demographics (N=83).

Variable		Message (n=46)	Video (n=37)	All
Age (years), mean (SD)		26.31 (8.80)	31.81 (13.89)	28.66 (11.48)
**Gender, n (%)**				
	Female	29 (63.0)	22 (59.5)	51 (61.4)
	Male	5 (10.9)	4 (10.8)	9 (10.8)
	Nonbinary	1 (2.2)	0 (0.0)	1 (1.2)
**Race, n (%)**				
	Asian	8 (17.4)	2 (5.5)	10 (12.0)
	Black	5 (10.9)	2 (5.5)	7 (8.4)
	Multiracial	2 (4.3)	1 (2.7)	3 (3.6)
	Native American	1 (2.2)	0 (0.0)	1 (1.2)
	White	12 (26.1)	16 (43.2)	28 (33.7)
**Ethnicity, n (%)**				
	Hispanic	10 (15.2)	7 (18.9)	17 (20.5)
	Non-Hispanic	24 (52.2)	18 (48.6)	42 (50.6)
**Income (US $), n (%)**				
	<25,000	20 (43.5)	14 (37.8)	34 (41.0)
	25,000-49,999	8 (17.4)	5 (13.5)	13 (15.7)
	50,000-74,999	3 (6.5)	4 (10.8)	7 (8.4)
	75,000-99,999	3 (6.5)	0 (0.0)	3 (3.6)
	≥100,000	1 (2.2)	1 (2.7)	2 (2.4)
Education (years), mean (SD)		14.43 (2.25)	15.68 (2.15)	14.95 (2.28)

### Treatment Outcome

Participants’ treatment outcome scores at pretreatment, posttreatment, and 6-month follow-up are presented in Tables 2 and 3, and between-group comparisons for treatment outcome are presented in Table 4.

Between pretreatment and posttreatment, participants in both treatment conditions reported significant improvement in depression (message: mean 6.97, SD 6.72, Cohen *d*=1.04; video: mean 6.23, SD 6.22, Cohen *d*=1.00), anxiety (message: mean 3.48, SD 5.73, Cohen *d*=0.61; video: mean 3.55, SD 4.30, Cohen *d*=0.83), and functional impairment (message: mean 0.71, SD 1.07, Cohen *d*=0.66; video: mean 0.45, SD 0.91, Cohen *d*=0.50). Changes in depression (*t*_47.52_=0.42, *P*=.68, Cohen *d*=0.11), anxiety (*t*_50.81_=–0.04, *P*=.97, Cohen *d*=–0.01), and functional impairment (*t*_49.22_=0.93, *P*=.36, Cohen *d*=0.25) did not significantly differ between the treatment conditions.

Over the course of treatment, participants in both treatment conditions experienced a significant change in depression (message: *b*=–0.50, SE 0.10, *P*<.001; video: *b*=–0.53, SE 0.10, *P*<.001), anxiety (message: *b=*–0.29, SE 0.08, *P*<.001; video: *b=*–0.35, SE 0.08, *P*<.001), and functional impairment (message: *b=*–0.05, SE 0.01, *P*<.001; video: *b=*–0.04, SE 0.02, *P*=.01). Changes in depression (*t*_553_=0.25, *P*=.80, Cohen *d*=0.02), anxiety (*t*_539_=0.60, *P*=.55, Cohen *d*=0.05), and functional impairment (*t*_552_=–0.13, *P*=.90, Cohen *d*=–0.01) in each session did not significantly differ between the treatment conditions.

Between pretreatment and 6-month follow-up, participants in both treatment conditions reported significant improvement in depression (message: mean 5.73, SD 6.09, Cohen *d*=0.94; video: mean 7.59, SD 6.55, Cohen *d*=1.16), anxiety (message: mean 2.22, SD 5.39, Cohen *d*=0.41; video: mean 3.24, SD 4.75, Cohen *d*=0.68), and functional impairment (message: mean 0.64, SD 1.16, Cohen *d*=0.55; video: mean 0.65, SD 0.86, Cohen *d*=0.75). Changes in depression (*t*_32.55_=–0.93, *P*=.36, Cohen *d*=–0.30), anxiety (*t*_37.37_=–0.65, *P*=.52, Cohen *d*=–0.19), and functional impairment (*t*_41.12_=–0.01, *P*=.99, Cohen *d*=0.00) did not significantly differ between the treatment conditions.

**Figure 2:**
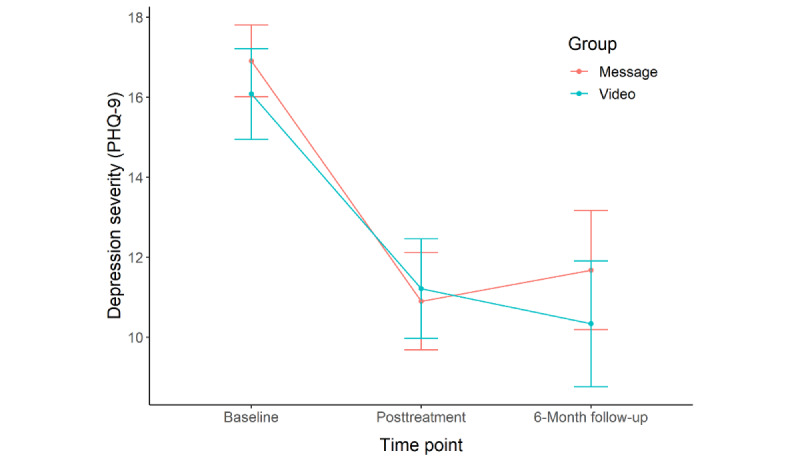
Comparison of depression severity between the 2 intervention conditions at pretreatment, posttreatment, and 6-month follow-up. PHQ-9: 9-item Patient Health Questionnaire.

**Figure 3:**
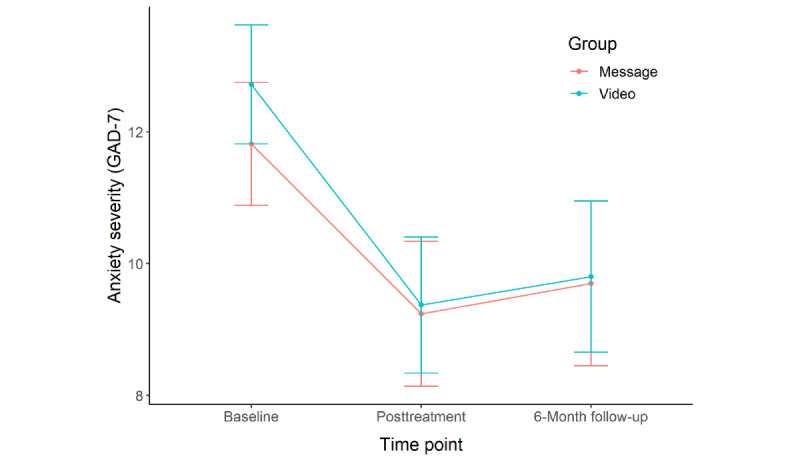
Comparison of anxiety severity between the 2 intervention conditions at pretreatment, posttreatment, and 6-month follow-up. GAD: 7-item Generalized Anxiety Scale.

**Figure 4:**
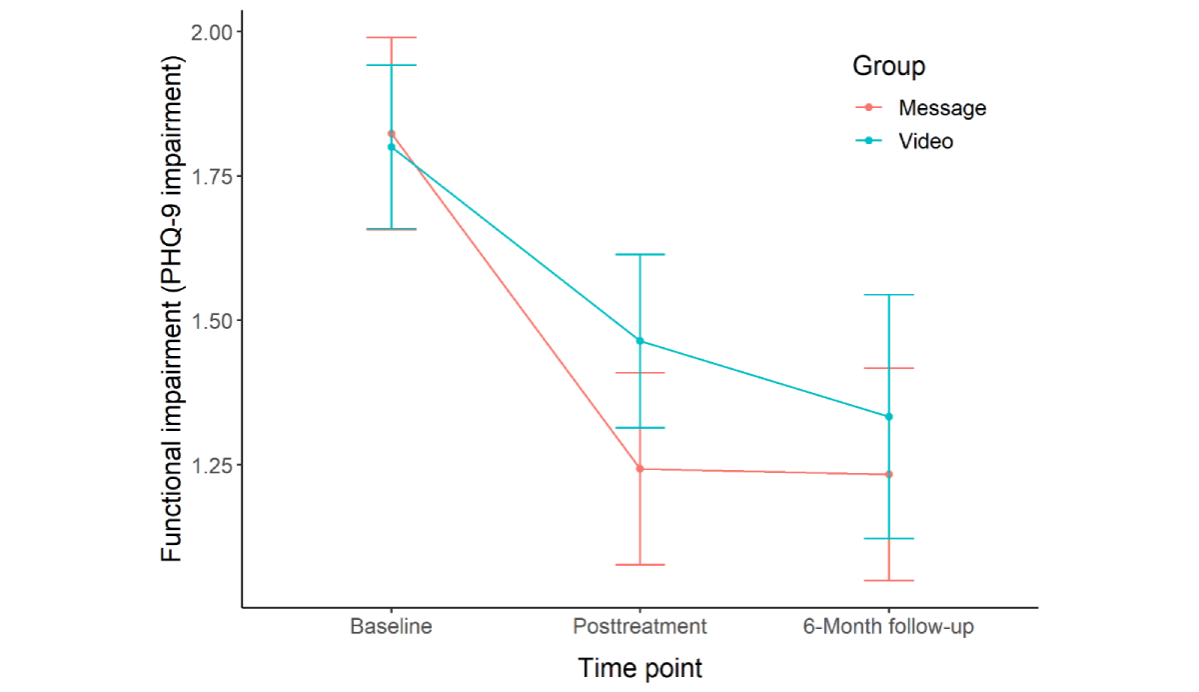
Comparison of functional impairment between the two intervention conditions at pretreatment, posttreatment, and 6-month follow-up. PHQ-9: 9-item Patient Health Questionnaire

Participants’ depression symptoms, anxiety symptoms, and functional impairment across pretreatment, posttreatment, and 6-month follow-up are shown in Figures 2-4, respectively.

**Table 2:** Means, SDs, and effect sizes for treatment outcome measures.

Outcome and condition		Pretreatment, mean (SD)	Posttreatment, mean (SD)	6-month follow-up, mean (SD)	Change per session, estimate (95% CI)
**Depression (0-27)**					
	Message	16.91 (5.14)	10.90 (7.58)	11.68 (7.88)	–0.50 (–0.69 to –0.31)
	Video	16.08 (5.67)	11.21 (6.58)	10.33 (7.21)	–0.53 (–0.74 to –0.33)
**Anxiety (0-21)**					
	Message	11.82 (5.36)	9.24 (6.78)	9.70 (6.87)	–0.29 (–0.45 to –0.12)
	Video	12.72 (4.51)	9.37 (5.36)	9.80 (5.14)	–0.35 (–0.51 to –0.19)
**Functional impairment (0-3)**					
	Message	1.82(0.97)	1.24 (1.01)	1.23 (1.01)	–0.05 (–0.08 to –0.02)
	Video	1.80 (0.71)	1.46 (0.79)	1.33 (0.97)	–0.04 (–0.08 to –0.01)

**Table 3:** With-group comparison for treatment outcome.

Outcome and condition		ΔPrepost			ΔPre-6-month follow-up	
		Mean (SD)	Cohen *d* (95% CI)	Mean (SD)		Cohen *d* (95% CI)
**Depression (0-27)**						
	Message	6.97 (6.72)	1.04 (0.60-1.46)	5.73 (6.09)		0.94 (0.47-1.40)
	Video	6.23 (6.22)	1.00 (0.48-1.51)	7.59 (6.55)		1.16 (0.53-1.77)
**Anxiety (0-21)**						
	Message	3.48 (5.73)	0.61 (0.22-0.99)	2.22 (5.39)		0.41 (0.01-0.80)
	Video	3.55 (4.30)	0.83 (0.33-1.30)	3.24 (4.75)		0.68 (0.14-1.20)
**Functional impairment (0-3)**						
	Message	0.71 (1.07)	0.66 (0.27-1.05)	0.64 (1.16)		0.55 (0.15-0.95)
	Video	0.45 (0.91)	0.50 (0.05-0.94)	0.65 (0.86)		0.75 (0.20-1.28)

**Table 4:** Between-group comparison for treatment outcome.

Variable		Message (n=46)	Video (n=37)	*t* test (*df*)	*P* value	Cohen *d* (95% CI)
**Change per session, estimate (SE)**						
	Depression (0-27)	–0.50 (0.10)	–0.53 (0.10)	0.25 (553)	.80	0.02 (–0.15 to 0.19)
	Anxiety (0-21)	–0.29 (0.08)	–0.35 (0.08)	0.60 (539)	.55	0.05 (–0.12 to 0.22)
	Functional impairment (0-3)	–0.05 (0.01)	–0.04 (0.02)	–0.13 (552)	.90	–0.01 (–0.18 to 0.16)
**ΔPrepost, mean (SD)**						
	Depression (0-27)	6.97 (6.72)	6.23 (6.22)	0.42 (47.52)	.68	0.11 (–0.43 to 0.66)
	Anxiety (0-21)	3.48 (5.73)	3.55 (4.30)	–0.04 (50.81)	.97	–0.01 (–0.56 to 0.53)
	Functional impairment (0-3)	0.71 (1.07)	0.45 (0.91)	0.93 (49.22)	.36	0.25 (–0.30 to 0.80)
**ΔPre-6-month follow-up, mean (SD)**						
	Depression (0-27)	5.73 (6.09)	7.59 (6.55)	–0.93 (32.55)	.36	–0.30 (–0.91 to 0.33)
	Anxiety (0-21)	2.22 (5.39)	3.24 (4.75)	–0.65 (37.37)	.52	–0.19 (–0.80 to 0.42)
	Functional impairment (0-3)	0.64 (1.16)	0.65 (0.86)	–0.01 (41.12)	.99	0.00 (–0.60 to 0.61)

### Treatment Acceptability

Between-group comparisons for treatment acceptability are presented in Table 5. Participants in both treatment conditions reported moderately high treatment credibility and expectancy (message: mean 26.74, SD 6.96; video: mean 27.41, SD 7.23) at pretreatment and working alliance with their treatment providers (message: 3.27, SD 0.98; video: 3.42, SD 0.86) at posttreatment. Treatment credibility and expectancy (*t*_44.29_=–0.34, *P*=.74, Cohen *d*=–0.09) and working alliance (*t*_41.50_=–0.52, *P*=.60, Cohen *d*=–0.15) did not significantly differ between the treatment conditions.

**Figure 5:**
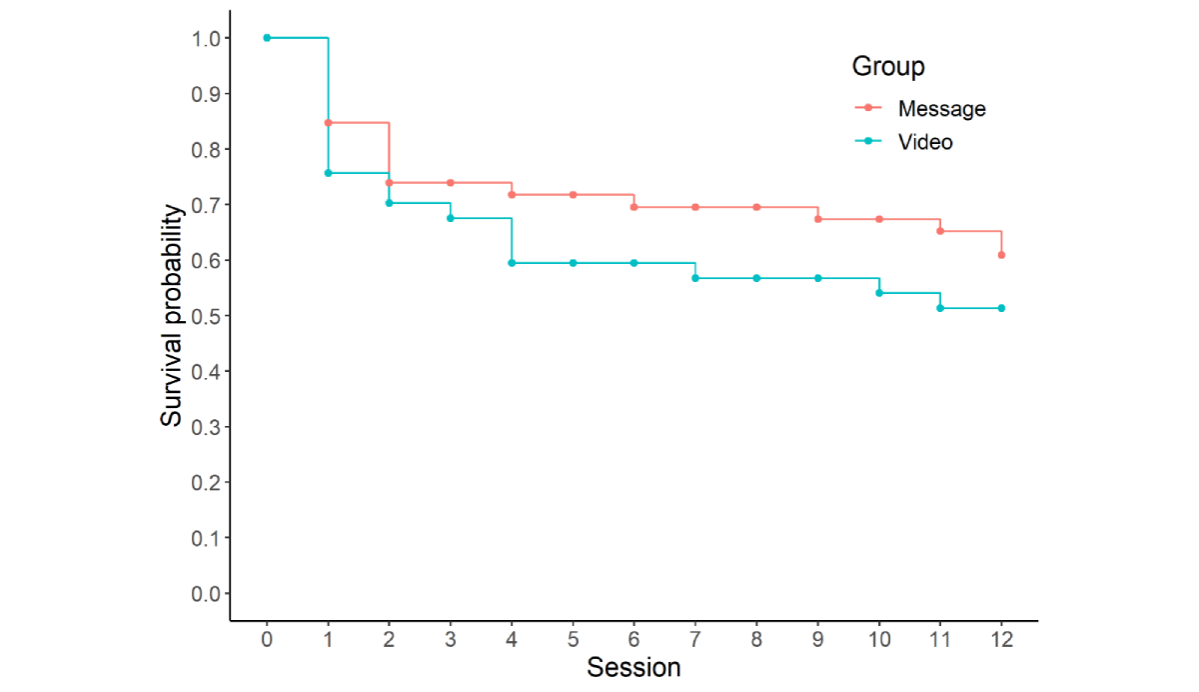
Survival curves of participant engagement for the 2 intervention conditions.

On average, participants in the message and video treatment conditions attended 8.46 (SD 5.14) and 7.19 (SD 5.46) sessions, respectively. The average number of sessions attended by the participants did not significantly differ between the treatment conditions (*t*_75.06_=1.08, *P*=.28, Cohen *d*=0.24). Survival curves for the 2 conditions are shown in Figure 5.

**Table 5:** Between-group comparison for treatment acceptability.

Variable	Message (n=46), mean (SD)	Video (n=37), mean (SD)	*t* test (*df*)	*P* value	Cohen *d* (95% CI)
Credibility (10-40)	26.74 (6.96)	27.41 (7.23)	–0.34 (44.29)	.74	–0.09 (–0.64 to 0.45)
Working alliance (0-5)	3.27 (0.98)	3.42 (0.86)	–0.52 (41.50)	.60	–0.15 (–0.75 to 0.44)
Engagement (0-12)	8.46 (5.14)	7.19 (5.46)	1.08 (75.06)	.28	0.24 (–0.20 to 0.67)

## Discussion

### Principal Results

This is one of the first studies to compare message-based care to the standard of care for the treatment of depression in a randomized clinical trial. We found that message-based care results in similar, positive outcomes to standard psychotherapy practice delivered over videoconferencing. Importantly, the magnitudes of change between pretreatment and posttreatment and between pretreatment and 6-month follow-up, as well as the rates of change over the course of treatment, were not smaller than those experienced by participants in the video-based treatment condition. Between pretreatment and 6-month follow-up, the effect sizes for message-based care were medium to large.

Importantly, not only did we find positive effects of message-based care on depression, anxiety, and functioning, but participants also found this method of care delivery to be credible and acceptable. There is considerable discussion in the field of digital mental health about the sporadic use of technology, where most people will use technology and quickly abandon it. Little is known as to the cause of this pattern of use, as assessments of credibility, acceptability, and utility are rarely collected. Our data find that, compared to what is evident in the use of digital mental health in research studies and the general community, these participants completed most sessions available to them and reported high levels of working alliance and engagement in the message-based treatment condition, comparable to those in the video-based treatment condition.

### Strengths and Limitations

This study has notable strengths. First, no study to date has evaluated the treatment outcome and acceptability of asynchronous message-based psychotherapy in a randomized controlled trial. Comparison to the more popular remote modality, video-based psychotherapy, is a critical step to creating and evaluating the evidence base for asynchronous message-based psychotherapy. Second, treatment outcome variables were collected longitudinally and allowed for both between-group and within-group comparisons. Within-group analyses were used to demonstrate significant changes in the outcome variables in both treatment conditions, and between-group analyses were used to demonstrate the noninferiority of asynchronous message-based psychotherapy to video-based psychotherapy. Third, participants were recruited from a neutral mental health advocacy organization, which increases the generalizability of the findings relative to recruiting individuals already interested in a particular platform or offering to begin.

Some limitations of this study also need to be noted. First, in both treatment conditions, there were participants who dropped out relatively early in the protocol. Still, on average, participants completed a considerable proportion of their treatment, and varying levels of treatment engagement are reflective of the psychotherapeutic treatments provided in community settings [38]. Future studies should consider investigating the predictors and impact of treatment engagement for asynchronous message-based psychotherapy in a larger, thus more powered, sample. Second, the randomized controlled trial was limited by the relatively small number of participants in each treatment condition. Given the promising results, however, the noninferiority of asynchronous message-based psychotherapy is currently being replicated in a larger sample comparison of the 2 treatment conditions [39]. Third, therapists were provided brief training on evidence-based treatments for depression, including interpersonal psychotherapy [40], cognitive behavioral therapy [41], behavioral activation [42], and problem-solving therapy [43], but adherence to specific treatments was not collected. Future research should quantify the use of treatment types. However, due to the randomization within therapists, we do not expect treatment modality to be a large factor in the outcome for each condition.

### Conclusions

This randomized controlled trial supports the treatment efficacy and acceptability of asynchronous message-based psychotherapy for major depressive disorder. Message-based psychotherapy also appears to be noninferior to the more widely accepted remote modality of video-based psychotherapy. Given its lower burden and greater accessibility compared to traditional psychotherapy, asynchronous message-based psychotherapy holds a promising avenue for future research and implementation.

## Data Availability

The data sets generated or analyzed during this study are not publicly available because no such consent to publicly share deidentified data was obtained from the study participants. They are, however, available from the corresponding author upon reasonable request with a fully executed Data Use Agreement.

## Appendix

Multimedia Appendix 1 CONSORT-eHEALTH checklist (V 1.6.1).

## Abbreviations

CONSORT: Consolidated Standards of Reporting Trials
GAD-7: 7-item Generalized Anxiety Scale
HIPAA: Health Insurance Portability and Accountability Act
PHQ-9: 9-item Patient Health Questionnaire
TRS: Expectations about Treatment/Treatment Rationale Scale
WAI-SR: Working Alliance Inventory—Short Revised

## References

[1] (2013). Diagnostic and Statistical Manual of Mental Disorders, 5th Edition.

[2] GBD 2019 Diseases Injuries Collaborators (2020). Global burden of 369 diseases and injuries in 204 countries and territories, 1990-2019: a systematic analysis for the global burden of disease study 2019. Lancet.

[3] Daly M, Sutin AR, Robinson E (2021). Depression reported by US adults in 2017-2018 and March and April 2020. J Affect Disord.

[4] Ettman CK, Abdalla SM, Cohen GH, Sampson L, Vivier PM, Galea S (2020). Prevalence of depression symptoms in US adults before and during the COVID-19 pandemic. JAMA Netw Open.

[5] Shigekawa E, Fix M, Corbett G, Roby DH, Coffman J (2018). The current state of telehealth evidence: a rapid review. Health Aff (Millwood).

[6] Perle JG, Nierenberg B (2013). How psychological telehealth can alleviate society's mental health burden: a literature review. J Technol Hum Serv.

[7] Carlo AD, Ghomi RH, Renn BN, Areán PA (2019). By the numbers: ratings and utilization of behavioral health mobile applications. NPJ Digit Med.

[8] Pierce BS, Perrin PB, Tyler CM, McKee GB, Watson JD (2021). The COVID-19 telepsychology revolution: a national study of pandemic-based changes in U.S. mental health care delivery. Am Psychol.

[9] Woerner M, Sams N, Nales CR, Gorstein T, Johnson M, Mosser BA, Areán PA (2022). Generational perspectives on technology's role in mental health care: a survey of adults with lived mental health experience. Front Digit Health.

[10] Hull TD, Malgaroli M, Connolly PS, Feuerstein S, Simon NM (2020). Two-way messaging therapy for depression and anxiety: longitudinal response trajectories. BMC Psychiatry.

[11] Chen L, Ingram D (2015). QuickStats: age-adjusted rates for suicide,* by urbanization of county of residence† — United States, 2004 and 2013. MMWR Morb Mortal Wkly Rep.

[12] Morales DA, Barksdale CL, Beckel-Mitchener AC (2020). A call to action to address rural mental health disparities. J Clin Transl Sci.

[13] Hollan JM, Bowling W, Reese RJ, Redmayne K, Clements-Hickman A, Leibowitz N, Hull TD (2021). Two-way messaging for rural users: a cohort comparison study. J Rural Ment Health.

[14] Ehlman DC, Yard E, Stone DM, Jones CM, Mack KA (2022). Changes in suicide rates - United States, 2019 and 2020. MMWR Morb Mortal Wkly Rep.

[15] Chan S, Li L, Torous J, Gratzer D, Yellowlees PM (2018). Review of use of asynchronous technologies incorporated in mental health care. Curr Psychiatry Rep.

[16] Reynolds DJ, Stiles WB, Bailer AJ, Hughes MR (2013). Impact of exchanges and client-therapist alliance in online-text psychotherapy. Cyberpsychol Behav Soc Netw.

[17] Hull TD, Mahan K (2017). A study of asynchronous mobile-enabled SMS text psychotherapy. Telemed J E Health.

[18] Broom MA, Ladley AS, Rhyne EA, Halloran DR (2015). Feasibility and perception of using text messages as an adjunct therapy for low-income, minority mothers with postpartum depression. JMIR Ment Health.

[19] DellaCrosse M, Mahan K, Hull TD (2018). The effect of messaging therapy for depression and anxiety on employee productivity. J Technol Behav Sci.

[20] Mental Health America.

[21] Talkspace.

[22] Harris PA, Taylor R, Minor BL, Elliott V, Fernandez M, O'Neal L, McLeod L, Delacqua G, Delacqua F, Kirby J, Duda SN, REDCap Consortium (2019). The REDCap consortium: building an international community of software platform partners. J Biomed Inform.

[23] Harris PA, Taylor R, Thielke R, Payne J, Gonzalez N, Conde JG (2009). Research electronic data capture (REDCap): a metadata-driven methodology and workflow process for providing translational research informatics support. J Biomed Inform.

[24] Kim J, Shin W (2014). How to do random allocation (randomization). Clin Orthop Surg.

[25] Kroenke K, Spitzer RL, Williams JB (2001). The PHQ-9: validity of a brief depression severity measure. J Gen Intern Med.

[26] Löwe B, Unützer J, Callahan CM, Perkins AJ, Kroenke K (2004). Monitoring depression treatment outcomes with the patient health questionnaire-9. Med Care.

[27] Spitzer RL, Kroenke K, Williams JBW, Löwe B (2006). A brief measure for assessing generalized anxiety disorder: the GAD-7. Arch Intern Med.

[28] Johnson SU, Ulvenes PG, Øktedalen T, Hoffart A (2019). Psychometric properties of the general anxiety disorder 7-item (GAD-7) scale in a heterogeneous psychiatric sample. Front Psychol.

[29] Collins J, Hyer L (1986). Treatment expectancy among psychiatric inpatients. J Clin Psychol.

[30] Chambless DL, Tran GQ, Glass CR (1997). Predictors of response to cognitive-behavioral group therapy for social phobia. J Anxiety Disord.

[31] Borkovec TD, Costello E (1993). Efficacy of applied relaxation and cognitive-behavioral therapy in the treatment of generalized anxiety disorder. J Consult Clin Psychol.

[32] Kirsch I, Henry D (1977). Extinction versus credibility in the desensitization of speech anxiety. J Consult Clin Psychol.

[33] Borkovec TD, Nau SD (1972). Credibility of analogue therapy rationales. J Behav Ther Exp Psychiatry.

[34] Devilly GJ, Borkovec TD (2000). Psychometric properties of the credibility/expectancy questionnaire. J Behav Ther Exp Psychiatry.

[35] Baier AL, Kline AC, Feeny NC (2020). Therapeutic alliance as a mediator of change: a systematic review and evaluation of research. Clin Psychol Rev.

[36] Hatcher RL, Gillaspy JA (2006). Development and validation of a revised short version of the working alliance inventory. Psychother Res.

[37] Munder T, Wilmers F, Leonhart R, Linster HW, Barth J (2010). Working alliance inventory-short revised (WAI-SR): psychometric properties in outpatients and inpatients. Clin Psychol Psychother.

[38] Cooper AA, Conklin LR (2015). Dropout from individual psychotherapy for major depression: a meta-analysis of randomized clinical trials. Clin Psychol Rev.

[39] Arean P, Hull D, Pullmann MD, Heagerty PJ (2021). Protocol for a sequential, multiple assignment, randomised trial to test the effectiveness of message-based psychotherapy for depression compared with telepsychotherapy. BMJ Open.

[40] Weissman MM, Markowitz JC, Klerman G (2008). Comprehensive Guide To Interpersonal Psychotherapy.

[41] Beck JS (2020). Cognitive Behavior Therapy: Basics and Beyond. 3rd Edition.

[42] Dimidjian S, Martell CR, Herman-Dunn R, Hubley S, Barlow DH (2014). Behavioral activation for depression. Clinical Handbook of Psychological Disorders: A Step-By-Step Treatment Manual. 5th Edition.

[43] Nezu AM, Nezu CM, D'Zurilla T (2012). Problem-Solving Therapy: A Treatment Manual.

